# Interspecies Transmission of Swine Influenza A Viruses and Human Seasonal Vaccine-Mediated Protection Investigated in Ferret Model

**DOI:** 10.3201/eid2909.230066

**Published:** 2023-09

**Authors:** Pauline M. van Diemen, Alexander M.P. Byrne, Andrew M. Ramsay, Samantha Watson, Alejandro Nunez, Ana v Moreno, Chiara Chiapponi, Emanuela Foni, Ian H. Brown, Sharon M. Brookes, Helen E. Everett

**Affiliations:** Animal and Plant Health Agency, Addlestone, UK (P.M. van Diemen, A.M.P. Byrne, A.M. Ramsay, S. Watson, A. Nunez, I.H. Brown, S.M. Brookes, H.E. Everett);; Istituto Zooprofilattico Sperimentale della Lombardia e dell'Emilia Romagna, Brescia, Italy (C. Chiapponi, E. Foni, A. Moreno)

**Keywords:** influenza, influenza A virus, zoonoses, vaccines, pig, ferret, viruses, respiratory infections

## Abstract

We investigated the infection dynamics of 2 influenza A(H1N1) virus isolates from the swine 1A.3.3.2 (pandemic 2009) and 1C (Eurasian, avian-like) lineages. The 1C-lineage virus, A/Pavia/65/2016, although phylogenetically related to swine-origin viruses, was isolated from a human clinical case. This strain infected ferrets, a human influenza model species, and could be transmitted by direct contact and, less efficiently, by airborne exposure. Infecting ferrets and pigs (the natural host) resulted in mild or inapparent clinical signs comparable to those observed with 1A.3.3.2-lineage swine-origin viruses. Both H1N1 viruses could infect pigs and were transmitted to cohoused ferrets. Ferrets vaccinated with a human 2016–17 seasonal influenza vaccine were protected against infection with the antigenically matched 1A pandemic 2009 virus but not against the swine-lineage 1C virus. Our results reaffirm the need for continuous influenza A virus surveillance in pigs and identification of candidate human vaccine viruses.

Influenza A viruses (IAVs) have pandemic potential and remain a threat to human and animal health, mainly owing to their intrinsic ability to continually diversify and infect a broad range of host species. Genetic heterogeneity in the 8-segmented IAV genome arises from the gradual accumulation of mutations (drift) owing to the low fidelity of the viral RNA polymerase. In addition, sporadic IAV gene segment exchange (shift) events can lead to the emergence of reassortant viruses with novel gene constellations and functional attributes. IAVs are characterized antigenically based on the hemagglutinin (HA) and neuraminidase (NA) envelope glycoproteins; HA incorporates the major epitopes conferring protective immunity (*1*–*3*).

Pigs are a key intermediate host species for IAV diversification; their susceptibility to IAVs originating from numerous mammalian (including human) and avian hosts enables virus reassortment and contributes to the expanding genetic heterogeneity of circulating swine IAV (swIAV) lineages (*2*,*4*,*5*). Because of this genetic heterogeneity, swIAVs are categorized according to HA phylogeny (*6*). Predominant viruses detected globally in swine populations belong to 3 main H1 genetic lineages (1A, 1B, and 1C) and multiple H3 clades, although antigenic and genetic differences might occur within these groupings according to geographic location (*6*,*7*). In Europe, the Eurasian avian-like H1 1C lineage (formerly termed the H1avN1 clade) has been enzootic in swine since the 1970s; phylogenetic evidence suggests direct incursion of an avian (duck)–origin virus into pigs (*8*). Subsequent reassortment with human-origin viruses produced the human-like H1 1B lineage (formerly known as the H1huN2 clade) and human-like H3N2 clades that cocirculated in pig populations in Europe as antigenically distinct IAV lineages until the introduction of the H1 1A.3.3.2 pandemic H1N1 (H1pdmN1) lineage in 2009 (*9**–**12*). The 1A.3.3.2 lineage continues to circulate and adapt in both pig and human populations globally and, in the swine reservoir, genetic mutation together with reassortment with established lineages is driving the expansion of viral diversity (*1*,*7*,*13*). This diversification of swIAVs circulating in pigs, combined with occasional transmission across the species barrier followed by host adaptation and escape from previous immunity, further elevates potential pandemic risk (*1*,*3*) and presents a challenge for disease control.

Sporadic human infection with so-called variant (v) influenza viruses that normally circulate in swine continue to be reported (*3*,*13*). In recent years, H1N1v infections with H1 1C swIAVs have been characterized in Europe (*14*–*18*) and Asia (*19*,*20*), and zoonotic infections caused by reassortant viruses incorporating gene segments from those 1C lineages have also been described (*21*–*27*). Experimental data indicate that some isolates have increased virulence profiles (*20*,*26*–*29*). Variant cases are frequently linked to persons or their contacts who have occupational exposure to pigs or exposure at animal exhibits. Onward transmission, assessed serologically, is reportedly limited or does not occur.

Vaccination remains the primary approach used to mitigate the disease burden of seasonal influenza in the human population and is the main defense against emergent IAVs with pandemic and epidemic potential, which occurred most recently in 2009. The most widely used human season influenza vaccines are trivalent or quadrivalent and contain inactivated antigens from 2 IAV subtypes (H1 and H3) and 1 or 2 influenza B virus lineages. However, because of constant antigenic change, contemporary IAVs are assessed biannually for antigenic match with vaccine antigens at the World Health Organization Vaccine Candidate Meeting, and candidate vaccine virus (CVV) recommendations are provided. The increased diversification of swIAVs and reports of zoonotic transmission have necessitated additional assessment of the antigenic match between CVVs and variant viruses and recommendation of swine-origin CVVs by OFFLU, the global network of expertise on animal influenza, should rapid vaccine antigen update be required (*1*,*30*).

We used the well-established ferret model of human influenza infection (*31*–*33*) to investigate 2 H1N1 viruses. The first virus was a 1A.3.3.2 lineage, swine-origin virus, A/swine/England/1353/2009 (*34*), incorporating all gene segments highly homologous to 2009 pandemic strains isolated from humans and swine (*9*). The second virus was a 1C.2.1 lineage virus, A/Pavia/65/2016 (*15*), which was associated with a human clinical case of influenza, but phylogenetic analysis confirmed that all gene segments were derived from contemporary 1C.2.1 viruses circulating in swine herds in Italy. We first assessed the ability of this virus to infect ferrets and undergo onward ferret-to-ferret transmission by direct or airborne exposure. We then evaluated the zoonotic potential of this 1C.2.1 virus by assessing transmission from infected pigs to cohoused ferrets and compared this virus in parallel with the swine-origin 1A.3.2.2 strain. Because swIAVs exhibit a higher degree of genetic and antigenic diversity than IAVs circulating in the human population at any one time, we also used the ferret model to investigate whether the human 2016–17 seasonal influenza vaccine could provide immune protection against the 2 swIAV strains.

## Materials and Methods

### Ethics Statement

We conducted in vivo studies at the Animal and Plant Health Agency (APHA), Addlestone, UK, in accordance with the Animal (Scientific Procedures) Act (ASPA) 1986 under license 70–8329 and approved by the APHA Ethical Review Panel. Results are reported according to the ARRIVE guidelines (*35*).

### Vaccines and Viruses

We immunized ferrets with a 2016–17 Northern Hemisphere seasonal influenza vaccine (Agrippal; CSL Seqirus, https://www.csl.com) that incorporated 3 inactivated virus antigens, A/California/7/2009 from the 1A.3.3.2 pandemic 2009 lineage (H1pdmN1), A/Hong Kong/4801/2014 (H3N2), and B/Brisbane/60/2008 (B/Victoria lineage). The H1N1 challenge strains were the 1A.3.3.2 (H1pdmN1) swine-origin virus A/swine/England/1353/2009 (*34*) and the 1C.2.1 (H1avN1) virus, A/Pavia/65/2016 (*15*). We propagated virus stocks in cell culture or specific-pathogen-free embryonated chicken eggs (Appendix). For consistency, we used the standard MDCK cell line for propagation and 50% tissue culture infectious dose (TCID_50_) titration of the inoculum for both virus strains (*36*).

### Animals

We conducted the studies using 34 three-month-old male ferrets from a registered breeder and 10 six-week-old Landrace cross male pigs from a commercial, high–health status herd. All animals were negative for influenza A virus infection, as determined by absence of viral RNA in nasal samples using real-time quantitative reverse transcription PCR (*37*) and swIAV-specific antibodies by hemagglutination inhibition (HI) assays against 4 antigens (*38*) and ID Screen competitive ELISA (Innovative Diagnostics, https://www.innovative-diagnostics.com) recognizing the viral nucleoprotein (NP) (Appendix). All ferrets and pigs were implanted with a subcutaneous biothermal IDentiChip (Destron Fearing, http://destronfearing.com) to monitor temperature. We monitored clinical signs, such as demeanor, appetite, temperature, and respiratory signs (e.g., coughing and sneezing), daily using a clinical scoring system designed for each species (Appendix Tables 1–4). We used a single subcutaneous injection of medetomidine (0.04 mg/kg; Vetoquinol, https://www.vetoquinolusa.com) and butorphanol (0.1 mg/kg; MSD Animal Health, https://www.msd-animal-health.com) to place ferrets under general anesthesia for virus inoculation and blood sample collection. We reversed the medetomidine anesthesia with a subcutaneous injection of atipamezole hydrochloride (0.4 mg/kg; Vetoquinol). We humanely killed animals by intravenous injection with pentobarbital sodium at study end.

### Study Design

**Figure 1:**
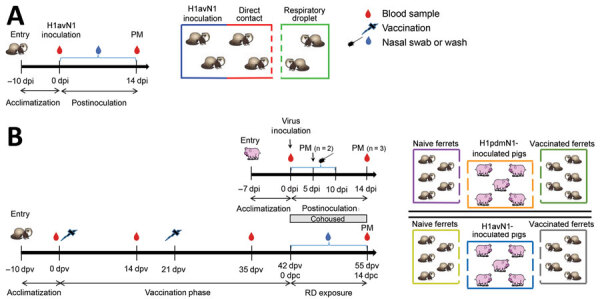
Outlines of 2 studies using ferret model to investigate interspecies transmission of swine influenza A viruses and human seasonal vaccine-mediated protection. A) Study 1 investigated the transmission ability of the A/Pavia/65/2016 (H1avN1) isolate in the ferret model of human infection. In 2 replicates, ferrets (n = 2) were intranasally inoculated and then cohoused with ferrets in direct contact (n = 2) and another group of ferrets (n = 2) separated by a perforated double divider to enable airborne exposure to respiratory droplets. B) Study 2 assessed airborne respiratory droplet transmission of 2 viruses from pigs to ferrets. In separate rooms, 2 groups of pigs (n = 5) were inoculated with either A/Pavia/65/2016 H1avN1 or A/swine/England/1353/2009 (H1pdmN1) virus and cohoused with naive (n = 5) and human seasonal 2016–17 influenza vaccine prime-boost–vaccinated ferrets (n = 5). Symbols on the timeline represent samples taken. dpc, days postcontact; dpi, days postinoculation; dpv, days postvaccination; PM, postmortem examination; RD, respiratory droplet.

The first study assessed the ability of the H1avN1 isolate, A/Pavia/65/2016, to infect ferrets and transmit to other ferrets by direct or airborne exposure (Figure 1, panel A). We randomly divided 12 male ferrets into 2 groups (n = 6). In each group, 2 animals were inoculated by intranasal instillation of 2 × 10^5^ TCID_50_ of strain A/Pavia/65/2016 in 0.5 mL (0.25 mL per nostril) and cohoused them with 2 ferrets in direct contact; we housed 2 additional ferrets in an adjacent cage separated by a perforated double divider to enable airborne exposure to respiratory droplets without nose-to-nose contact. We collected nasal wash samples daily from alert ferrets and took blood samples (clotted) from the jugular vein of anesthetized ferrets before inoculation and at study completion (14 days postinoculation [dpi]).

The second study (Figure 1, panel B) evaluated the infection dynamics of the H1avN1 and H1pdmN1 viruses in pigs and assessed the interspecies transmission of these viruses from pigs to vaccinated or naive ferrets. We randomly distributed 20 male ferrets into 4 groups (n = 5), then prime-boost vaccinated 2 groups with 1 dose (0.5 mL) of human seasonal vaccine administered by intramuscular injection into the thigh muscle at a 21-day interval. Two unvaccinated ferrets were not virus exposed and were housed separately to serve as negative control animals. Blood samples (clotted and heparin anticoagulated) were taken under anesthesia from the jugular vein of all ferrets before first vaccination, before boost, at 35 days after vaccination, and at study completion.

We randomly assigned pigs to 2 groups (n = 5) and housed them in separate rooms for inoculation with either 4 × 10^6^ TCID_50_ A/swine/England/1353/2009 or 2 × 10^6^ TCID_50_ A/Pavia/65/2016 virus stocks in 4 mL (2 mL/nostril) using a MAD Nasal Intranasal Mucosal Atomization Device (Teleflex, https://www.teleflex.com). After 24 hours (corresponding to 42 days after vaccination), we cohoused a vaccinated and a nonvaccinated ferret group (each n = 5) in each pig room to enable airborne respiratory droplet exposure in the shared airspace. We obtained nasal swab (pig) and wash (ferret) samples daily from alert animals after inoculation or contact to monitor viral shedding. We obtained blood samples (clotted and heparin anticoagulated) from the left jugular vein of pigs before inoculation and before euthanasia. At 5 dpi, we humanely killed 2 pigs from each group to perform postmortem examination. We humanely killed the remaining pigs at 14 dpi when virus shedding had ceased; we humanely killed ferrets at 14 dpc.

### Sample Analysis

We performed sample processing as described (Appendix). To monitor nasal shedding of viral RNA, we extracted nasal samples using a QIAamp Viral RNA Biorobot Kit (QIAGEN, https://www.qiagen.com) and quantified the RNA present using real-time quantitative reverse transcription PCR directed against the matrix (M) gene (*37*). Viral RNA quantity is expressed as relative equivalent units (REUs) per milliliter and was evaluated according to a standard 10-fold dilution series of RNA prepared from each challenge virus stock, with known TCID_50_ titer. REUs provide a relative quantification of infectious virus present, inferred from the linear, proportional relationship between viral infectivity and viral RNA quantity standardized by volume of nasal sample (*38*), and enables rapid, sensitive, and direct analysis of clinical samples. We evaluated the humoral response as described (Appendix). We used a commercial, competitive ELISA (IDVet, Innovative Diagnostics) to detect NP-specific antibodies. The competition percent is calculated as (OD_sample_/OD_negative_) × 100% and is expressed as the inverse, such that results <50% are considered negative. We used HI and virus neutralization assays (*39*,*40*) to evaluate antibody titers elicited by the challenge virus for each group. To monitor the cellular response, we assessed interferon-γ–producing peripheral blood mononuclear cells using the ELISpot assay (Appendix). During the postmortem at 5 dpi, we collected pig respiratory tissues (nasal turbinate, trachea, and lung) in 10% (vol/vol) phosphate-buffered formalin for immunohistochemical analysis of NP to assess viral distribution (Appendix) (*41*).

### Statistical Analysis

We performed statistical analyses using GraphPad Prism7 (GraphPad, https://www.graphpad.com) to calculate arithmetic and geometric means, associated standard deviation or error of the mean, analysis of variance, and associated post-hoc Tukey tests. Titer and REU values were logarithmically transformed. We used a 2-way repeated measures analysis of variance to analyze repeated measurements such as viral RNA quantity and immune response values. We identified statistically significant differences using the Tukey multiple comparisons test and considered results significant when p<0.05.

## Results

**Figure 2:**
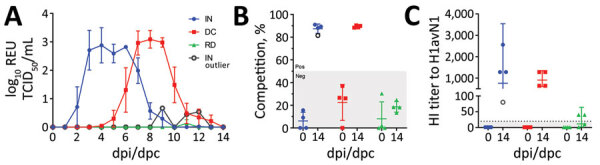
Longitudinal monitoring of A/Pavia/65/2016 influenza A virus infection and transmission in ferrets. In 2 replicates, ferrets (n = 2) were intranasally inoculated with the A/Pavia/65/2016 strain and cohoused with ferrets in direct contact (n = 2) or in the same airspace (n = 2) enabling airborne exposure to respiratory droplets. Infection was evaluated by (A) monitoring daily nasal shedding of viral RNA between 0–14 dpi expressed as REUs. The specific humoral immune response was evaluated at 0 and 14 dpi using (B) a competitive ELISA to determine nucleoprotein-specific antibody titer, expressed as the inverse of the competition percentage (%) or (C) HI titer with the homologous virus. Competition percentage was calculated as (1 – sample/negative) ´ 100. Nucleoprotein competition percentage <50% or HI titer of <20 are considered negative (gray shaded areas). Outlier results for a single ferret in the intranasally inoculated group (IN-outlier) were excluded from the analysis and are shown by hollow black symbols. DC, direct contact; dpc, days postcontact; dpi, days postinoculation; HI, hemagglutination inhibition; IN, intranasally inoculated; Neg, negative; Pos, positive; RD, respiratory droplet; REU, relative equivalent unit; TCID_50_, 50% tissue culture infectious dose.

Intranasal inoculation of ferrets with the A/Pavia/65/2016 isolate in study 1 (Figure 1, panel A) resulted in productive infection (Figure 2, panels A–C), as revealed by nasal shedding of viral RNA detected at 2–8 dpi and seroconversion by 14 dpi, evaluated by NP ELISA and HI assays. Clinical signs, such as demeanor, appetite, temperature, and respiratory signs (e.g., coughing and sneezing), were normal/not apparent or mild and did not exceed a total score of 4 for any individual ferret (Appendix Tables 1–2). One of 4 ferrets did not shed viral RNA after direct inoculation, although seroconversion was detected by NP ELISA and HI assays, indicating immune exposure to virus. This observation could reflect differences in the susceptibility of a genetically outbred ferret population or experimental variation. Cohoused ferrets also demonstrated evidence of productive infection, indicating virus transmission by direct contact. Viral transmission by the airborne route was not detected. However, respiratory droplet exposure did elicit an antibody response in some ferrets that was at or below the lower limit of detection of the assays, possibly indicating immune exposure. Those results indicated that ferrets were a suitable challenge model for the A/Pavia/65/2016 H1avN1 isolate.

In study 2 (Figure 1, panel B), 10 ferrets were prime-boost vaccinated with a trivalent human influenza vaccine from the 2016–17 season; 10 ferrets were not vaccinated to serve as naive control animals. The interval between prime and boost vaccinations was 3 weeks, and the vaccination phase continued for a further 3 weeks. We then housed 2 groups of pigs (n = 5) in separate rooms and inoculated with either the 1A.3.2.2 swine-origin (H1pdmN1) virus A/swine/England/1353/2009 (room 1) or the 1C.2.1 human isolate (H1avN1) virus, A/Pavia/65/2016 (room 2). We then cohoused a group of naive ferrets (n = 5) and a group of vaccinated ferrets (n = 5), held in separate cages, with the infected pigs in each room. We monitored all animals daily; clinical signs were mild or absent according to the clinical scoring systems for ferrets and pigs (Appendix Tables 1–4), indicating that both virus strains had similar, mild pathogenesis profiles and infection was effectively resolved in both host species.

**Figure 3:**
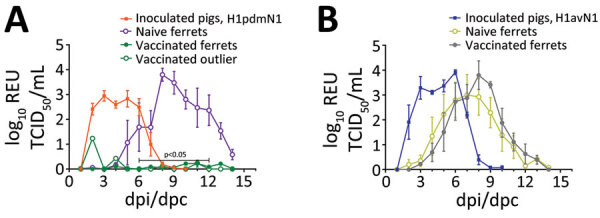
Nasal shedding of viral RNA monitored in pigs intranasally inoculated with influenza A virus strains A/swine/England/1353/2009 (H1pdmN1) (A) or A/Pavia/65/2016 (H1avN1) (B) and in naive or vaccinated ferrets cohoused in the same airspace as inoculated pigs. Viral RNA was quantified by real-time quantitative reverse transcription PCR in longitudinal nasal samples collected daily until 14 dpi (pigs) or 14 dpc (ferrets) and is expressed as REU based on an RNA quantification standard prepared from the corresponding virus stock. In vaccinated ferrets (n = 4) exposed to the H1pdmN1 strain, nasal shedding of viral RNA between 6 dpc and 12 dpc was significantly different from the naive ferret group (p<0.05). Results for the remaining ferret in this group are shown as outlier data (hollow green circles). dpc, days postcontact; dpi, days postinoculation; REU, relative equivalent unit; TCID_50_, 50% tissue culture infectious dose.

We quantified viral RNA in daily nasal samples to assess virus shedding (Figure 3, panels A, B). In pigs, nasal shedding of viral RNA peaked at 2–6 dpi and ceased by 8 dpi, indicating that both virus strains caused a productive infection that resolved quickly. We detected viral RNA in nasal wash samples collected from all naive, unvaccinated ferrets as well as in samples collected from vaccinated ferrets that had been exposed to the 1C.2.1 virus. Conversely, the ferret group that had received the human seasonal vaccine and was then exposed to the swine-origin 1A.3.2.2 virus (Figure 3, panel A) showed a significant reduction in viral shedding in nasal samples. A single ferret in this vaccinated group showed an outlier response of transient, low level of viral RNA shedding on nonconsecutive days. Taken together, those shedding profiles indicated that both viruses could be transmitted from infected pigs to naive ferrets by the airborne route and cause productive infection. In addition, the human seasonal vaccine could only elicit protective immunity against an antigenically similar challenge virus, namely the swine-origin 1A.3.3.2 virus but not the antigenically distinct 1C.2.1 virus.

**Figure 4:**
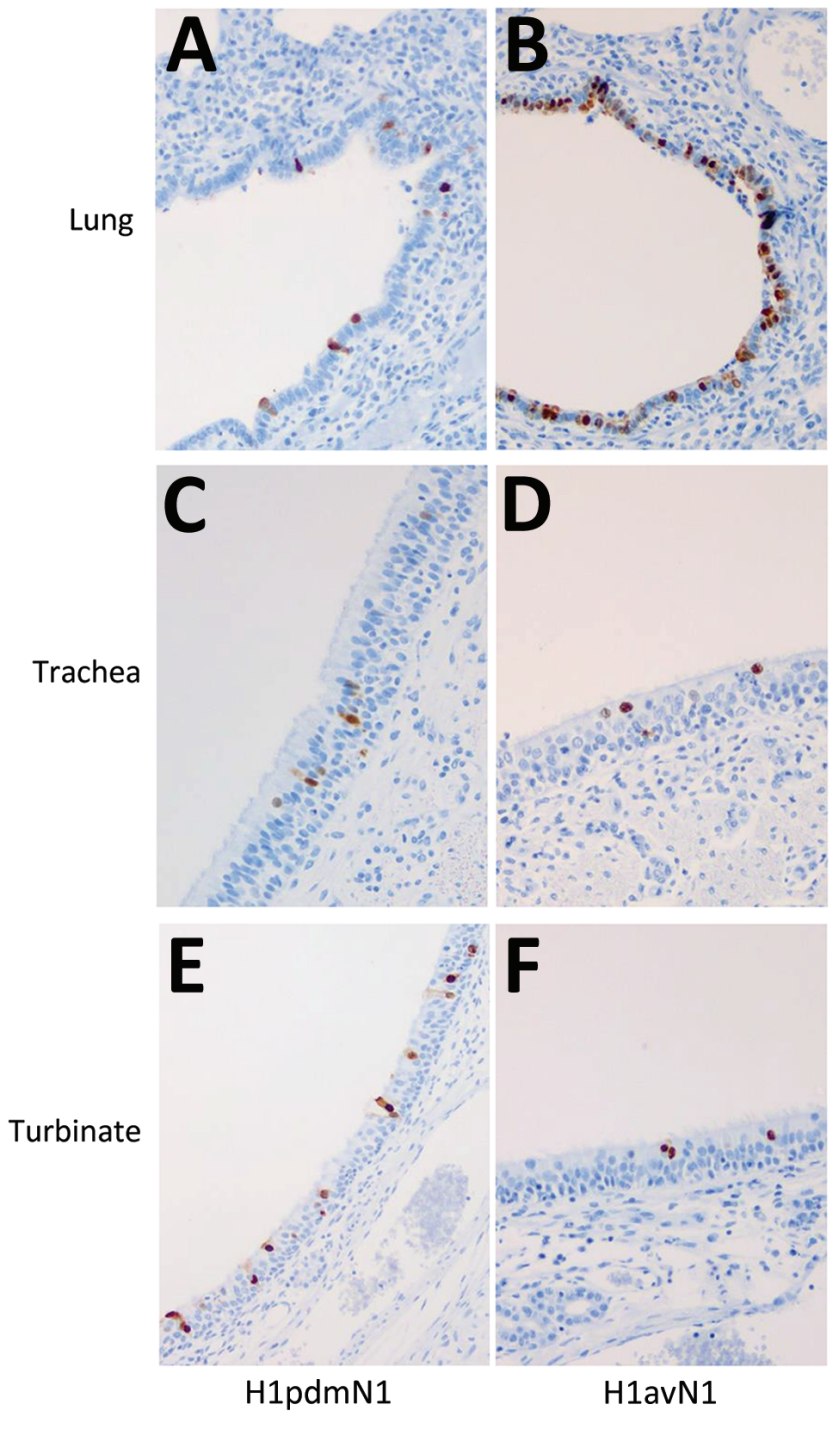
Immunohistochemical detection of viral nucleoprotein in pig tissues. Immunolabelling of influenza A viral nucleoprotein in respiratory tissues collected from pigs at 5 dpi after inoculation with A/swine/England/1353/2009 (H1pdmN1; panels A, C, and E) or A/Pavia/65/2016 (H1avN1; panels B, D, and F) viruses reveals presence of viral nucleoprotein antigen (brown staining) in respiratory epithelial cells of the lung, trachea, and nasal turbinate for both viruses. Original magnification × 400.

**Figure 5:**
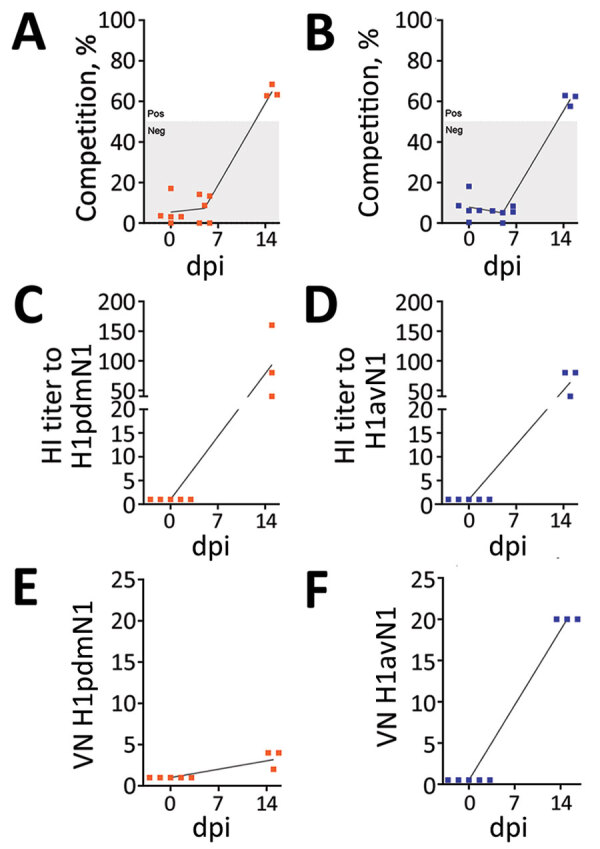
Characterization of virus-specific humoral responses following intranasal inoculation of pigs with A/swine/England/1353/2009 (H1pdmN1, panels A, C, E) or A/Pavia/65/2016 (H1avN1, panels B, D, F). Antibody titers were monitored at 0, 5, and 14 dpi by nucleoprotein competitive ELISA (A, B) and are expressed as competition percentage and considered negative if <50% (gray area). Competition percentage was calculated as (1 – sample/negative) × 100. Hemagglutination inhibition (C, D) and virus neutralization (E, F) titers were assessed at 0 and 14 dpi using the homologous virus for each group. Both titers were normalized to the individual prevaccination titers (0 days postvaccination). dpi: days postinoculation

Immunohistochemical analysis of pig tissues collected at 5 dpi (Figure 4) demonstrated immunolabelling of viral NP antigen in the nucleus and cytoplasm of epithelial cells of the respiratory mucosae, including nasal turbinate, trachea, and bronchi and bronchioles in the lungs of pigs inoculated with either virus, indicating comparable replication of both virus strains. Specific humoral responses were detected in both groups of pigs at 14 dpi, indicated by the increase in NP (Figure 5, panels A, B) and HI (Figure 5, panels C, D) antibody titers. Furthermore, we detected a specific neutralizing antibody response (Figure 5, panels E, F) for each inoculated virus at 14 dpi, although titers were considerably lower in H1pdmN1 virus–infected pigs. Taken together, those results indicate that all pigs seroconverted after virus inoculation and that infections were productive.

**Figure 6:**
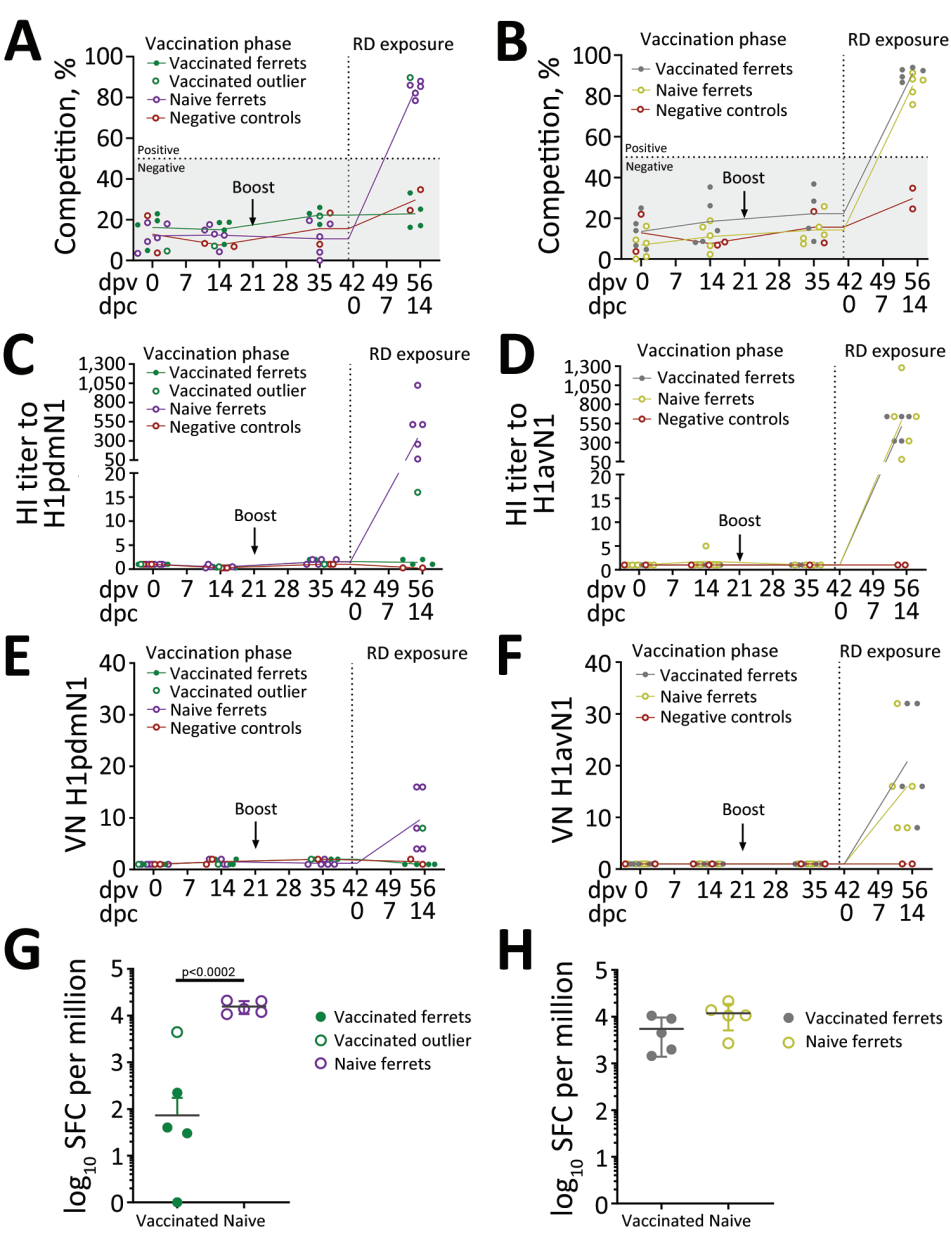
Immune parameters assessed in naive and vaccinated ferrets before and after exposure to pigs infected with influenza A viruses A/swine/England/1353/2009 (H1pdmN1, panels A, C, E, and G) or A/Pavia/65/2016 (H1avN1, panels B, D, F, and H). Data from a single outlier, a vaccinated ferret exposed to the H1pdmN1 virus, were excluded from analysis but are shown. Negative control ferrets (n = 2) were not vaccinated or exposed to infectious virus. Specific humoral responses were assessed longitudinally in serum. Antibody titers detected by NP competition ELISA (A, B) are expressed as competition percentage and considered negative if <50% (gray area). Competition percentage was calculated as (1 – sample/negative) × 100. HI (C, D) and VN (E, F) were determined using the homologous virus for each group. Both HI and VN titers are normalized to the individual prevaccination titers (0 dpv). ELISpot analysis (G, H) evaluated the number of interferon-γ–producing peripheral blood mononuclear cells induced by 18-mer nucleoprotein peptides, represented as per 1 million, at 14 dpc (RD exposure). dpv, days postvaccination; dpc, days postcontact; HI, hemagglutination inhibition; NP, nucleoprotein; RD, respiratory droplets; SPC, spot-forming cells; VN, virus neutralization.

Humoral immune responses in virus-exposed ferrets were evaluated by NP ELISA (Figure 6, panels A, B) as well as HI (Figure 6, panels C, D) and virus neutralization (Figure 6 panels, E, F), using the homologous viruses. Antibody responses to vaccination were low or undetectable. Unvaccinated ferrets in both groups seroconverted after virus exposure, as did vaccinated ferrets cohoused with pigs inoculated with the 1C.2.1 virus. In contrast, vaccinated ferrets cohoused with pigs infected with the swine-origin 1A.3.3.2 virus mounted no detectable influenza-specific humoral response, apart from the single ferret that showed transient, low-level nasal shedding (Figure 3, panel A). The humoral responses shown separately for this ferret as an outlier from the group data (Figure 6, panels A, C, E) could reflect differences in the immune response elicited by vaccination in this individual ferret, as observed in outbred populations. Two nonvaccinated, nonexposed negative control animals did not produce specific humoral immune responses, as was expected. ELISpot analysis (Figure 6, panels G, H) showed that infection elicited a detectable cellular response after stimulation with NP peptides, but it was considerably reduced (p<0.0002) in vaccinated ferrets exposed to the H1pdmN1 virus, although the single outlier ferret showed an intermediate response.

Collectively, those results indicate that naive ferrets became productively infected after airborne exposure to virus shed by infected pigs but nevertheless mounted an effective humoral and cellular response, resulting in resolution of infection. Conversely, productive infection did not occur in the 1A.3.3.2 H1N1–exposed ferrets with previous vaccine-mediated immunity when the vaccine antigen was well matched to the challenge strain, although we did not identify corresponding immune determinants. Vaccination did not prevent infection of ferrets with the 1C.2.1 virus. 

## Discussion

H1 1C Eurasian avian-like viruses have been circulating in swine herds in Europe for >40 years, most likely following direct introduction from an avian host into pigs (*8*). This virus clade remains a potential zoonotic risk, as highlighted by sporadic human H1N1v cases caused by this swIAV lineage and reassortant viruses, as well as by experimental data obtained using the ferret model (*20*,*24*,*26*,*28*,*29*).

The ferret is a robust animal model species for studying influenza arising from both human- and swine-origin IAV infections (*32*,*42*) and for studying influenza vaccines (*31*); we used that model to characterize the 1C.2.1 lineage virus, A/Pavia/65/2016. Virus infection transmitted effectively between ferrets by the direct contact route but not by airborne respiratory droplet exposure, suggesting that sustained transmission in human populations would be limited, as supported by epidemiologic findings (*15*). Of note, nasal shedding of virus by pigs resulted in respiratory droplet infection of susceptible, cohoused ferrets. We speculate that result occurred because of the larger volume of respiratory droplets exhaled by pigs, which have a larger lung volume than ferrets, thereby increasing the viral load. The virologic profile of the A/Pavia/65/2016 isolate, when compared in the same interspecies transmission model to A/swine/England/1353/2009, a swine-origin H1N1 virus from the 1A.3.3.2 lineage, demonstrated that all experimentally infected animals exhibited mild or no clinical signs of influenza, mounted an effective humoral and cellular immune response, and resolved the infection. Our findings therefore indicate that the A/Pavia/65/2016 strain does not have an increased pathogenicity profile compared to the 1A.3.3.2 strain when assessed in 2 animal models, as predicted from phylogenetic data, despite having originated from a human clinical case. In addition, our study reaffirms the value of the interspecies transmission model for assessing zoonotic potential (*20*,*38*,*42*–*45*).

We assessed immunity provided by the 2016–17 human seasonal influenza vaccine against the 2 swIAV isolates by cohousing naive and vaccinated ferret groups with pigs shedding the respective virus strains. All ferret groups, except the vaccinated ferrets exposed to the H1pdmN1 virus–infected pigs, had a viral nasal shedding profile consistent with productive infection and mounted a detectable humoral and cellular immune response. Conversely, nasal shedding in the vaccinated, 1A.3.3.2 H1N1–exposed ferret group was significantly reduced, suggesting that the human seasonal vaccine provided immune protection from infection by the antigenically matched swine-origin challenge strain. However, the immune response after infection was low in that ferret group, so the correlates of protection remain unknown. In both studies, individual ferrets in single groups displayed outlier responses to infection or vaccination, possibly reflecting the differences observed in outbred populations. 

Despite such limitations and the constraints of low group numbers, this study enabled effective modeling of interspecies transmission of influenza. The experimental design benefited from using pigs as a biological host for the virus strains studied. In addition, the study design provided a controlled and biologically relevant system to study interspecies airborne transmission to ferrets, a well-established animal model for human influenza; including naive and vaccinated ferret groups enabled modeling of human populations with varied prior immunity to influenza (*31*).

As part of the World Health Organization influenza pandemic preparedness initiative, CVVs for human seasonal vaccines are identified twice a year. Considering the increase in reports of zoonotic infections, OFFLU has contributed data for selecting swIAV-origin CVVs should a zoonotic spillover event necessitate a rapid update of human seasonal vaccine antigens. Within-clade diversity of 1C-lineage swIAVs hampers the selection of candidate antigens, as has also been observed for 1B viruses (*24*,*43*,*46*) and, despite the A/Pavia/65/2016 strain being in the same 1C2.1 genetic lineage as the CVV A/Netherlands/3315/2016, antigenic cross-reactivity is low (*1*). Those findings reinforce the need for continued CVV assessment for swIAVs to ensure pandemic preparedness. Furthermore, recent studies in the ferret model have demonstrated the potential for IAV and SARS-CoV-2 co-infection. Clinical severity was ameliorated by influenza vaccination, thereby demonstrating the potential importance of ensuring vaccine immunity to circulating influenza strains in the human population (*47*).

Our study confirms that vaccine and challenge strains must be antigenically matched to elicit vaccine-mediated protective immunity and that the immune status of the human population might not provide complete immunity to all currently circulating swine influenza A virus H1N1 strains. Continual evaluation and monitoring of IAVs circulating in human and swine populations is required to identify potential pandemic threats; broadly effective vaccines for both human and veterinary use are needed to mitigate these threats.

Appendix Additional information about interspecies transmission of swine influenza A viruses and human seasonal vaccine-mediated protection investigated in ferret model.
