# Supplementary material for: Interspecies Transmission of Swine Influenza A Viruses and Human Seasonal Vaccine-Mediated Protection Investigated in Ferret Model
Source: Emerg Infect Dis. 2023 Sep;29(9):1798–807. doi: 10.3201/eid2909.230066 (PMC10461666; doi:10.3201/eid2909.230066)
Supplement: Appendix — Additional information about interspecies transmission of swine influenza A viruses and human seasonal vaccine-mediated protection investigated in ferret model. [file 23-0066-Techapp-s1.pdf]

Article DOI: https://doi.org/10.3201/eid2909.230066

EID cannot ensure accessibility for supplementary materials supplied by authors. Readers who have difficulty accessing supplementary content should contact the authors for assistance.

Interspecies Transmission of Swine Influenza A Viruses and Human Seasonal Vaccine-Mediated Protection Investigated in Ferret Model

Appendix

Supplemental Methods

Virus passage history

Virus stocks were propagated in cell culture or SPF embryonated chicken eggs as described (*1*). The A/Pavia/65/2016 strain (*2*) was provided by the Istituto Zooprofilattico Sperimentale della Lombardia e dell'Emilia Romagna (IZSLER), Brescia. At APHA, this stock was propagated twice in SPF embryonated chicken eggs, once in the newborn pig trachea (NPTr) cell line and the inoculum was produced in MDCK cells. The A/swine/England/1353/2009 strain (*3*) isolated at APHA was propagated twice in SPF embryonated chicken eggs and the inoculum was produced in Madin-Darby canine kidney (MDCK) cells. Virus stocks prepared as reagents for serology assays were propagated in SPF embryonated chicken eggs.

Sample processing

Nasal samples were obtained daily post-infection without anesthesia and using appropriate restraint. Ferret nasal wash samples were obtained using 1ml Dulbecco’s PBS (DPBS, GIBCO) and stored at −80°C. Nasal swabs (MWE) obtained daily from pigs (one per nostril) were pooled and eluted in 1ml Leibovitz’s L-15 medium with L-glutamine supplemented with 100U/ml penicillin, 1000ug/ml streptomycin and 1% fetal bovine serum (all GIBCO). Samples were stored at −80°C.

Clotted whole blood was centrifuged at 1200 g for 10min at room temperature and serum was collected and stored at −20°C. PBMCs were isolated from heparin anticoagulated whole blood. Erythrocytes were lysed using ammonium chloride-based PharmLyse (BD) and cells were resuspended in complete RPMI (cRPMI) containing 10% heat inactivated FBS, 1% glutamine and 1% penicillin/streptomycin (all GIBCO). Cell density was adjusted to 1–2 × 10^7 cells/mL in cold 10% dimethylsulfoxide (DMSO) (Sigma-Aldrich) in Foetal Bovine Serum (FBS, Sigma-Aldrich) for cryopreservation.

Serology and Immunology analysis

ELISA assay.

Antibody titers specific for the influenza A virus nucleoprotein (NP) were evaluated using a competitive, multi-species ELISA (ID Screen® IDvet) according to the manufacturer's instructions. Dilutions of 1:40 and 1:10 were used for pig and ferret sera respectively. The competition percent is calculated as [(OD_{sample}/$OD_{negative}$) x 100%] and is expressed as the inverse, such that results below 50% are considered negative.

Haemagglutinin inhibition (HI) assay

HI antibody titers were determined against A/Pavia/65/2016 and/or A/swine/England/1353/2009 whole virus antigens propagated in SPF embryonated chicken eggs as described for pig and ferret sera (*4*). In short, 10-fold serial dilutions of receptor destroying enzyme (RDE)-treated serum were incubated with 4 hemagglutinating (HA) units of an influenza virus stock for 30 min at RT. After a further 1hr incubation with chicken erythrocytes, the HI titer was assessed as the highest serum dilution that prevented hemagglutination.

Virus neutralisation (VN) assay

VN assays were conducted as described (*5*) using the A/Pavia/65/2016 and A/swine/England/1353/2009 viruses. VN titers were expressed as the reciprocal of the highest dilution of serum where virus infection of cells was prevented.

ELISpot analysis

High Protein Binding Immobilon-P membrane plates (Millipore) were coated overnight with 0.5 mg/ml anti-ferret IFN-γ mAb (MabTech) at 4°C, washed and blocked. PBMC density was adjusted to 1x10^6/ml, and a 50 µl volume was added to each well. PBMC were stimulated

with 5 μg/ml of a pool of peptides representing NP which were 18aa in length and overlapped by 6aa (GL Biochem (Shanghai) Ltd). Pokeweed mitogen (PWM, Sigma-Aldrich) treatment was used as a positive control. Plates were incubated for 48h at 37°C, 5% CO_2. IFN-γ spot forming cells (SFC) were visualized by 2h incubation with biotinylated anti-ferret IFN-γ mAb (0.05 μg/ml, MabTech) followed by 1h incubation with streptavidin-Alkaline Phosphatase (0.5 μg/ml, MabTech) and color development with AP conjugate substrate kit (BioRad, UK) with washing steps between each incubation. SFC were evaluated using an automated ELISpot reader (AID) and Log_{10} SFC was used in statistical analysis because of the approximate log-normal distribution of ELISpot counts in animals (*6*).

Immunohistochemistry (IHC) analysis

Detection of IAV nucleoprotein (NP) by IHC was performed as described (*7*). Briefly, 4μm sections were dewaxed and endogenous peroxidase activity was quenched with a methanol/hydrogen peroxide block for 15min and treated with Protease XXIV for 10min at room temperature. Primary antibody cross-reactivity with tissue constituents was prevented using a normal immune serum block. Samples were incubated with an anti-influenza A nucleoprotein primary mouse monoclonal antibody (Statens Serum Institut) for 1 hour and Dako ENVISION polymer for 30min at room temperature. The immunohistochemical signal was visualized using 3,3-diaminobenzidine (Sigma-Aldrich) and counterstained in Mayer's haematoxylin (Surgipath).

Appendix Table 1. Clinical scoring scheme for ferrets infected with influenza A virus

Parameter	Criteria	Score*
Alertness	Attentive (curious, alert)	0
	Slightly reduced – hesitant, disinterested	1
	Inactive, gets up only when stimulated, lies down again	2
	Recumbent, does not get up when stimulated	3
Weight loss	No significant weight loss 0%–5%	0
	Weight loss 5%–10% from acclimatization baseline	1
	Weight loss 10%–15% from acclimatization baseline	2
	Weight loss over 15% from acclimatization baseline	3
Body tension	Relaxed	0
	Slightly hunched back	2
	Flaccid or Hunched and rigid body	3
Breathing	Normal	0
	Sneezing / coughing	1
	Intermittent breathing difficulties or increase/decrease in respiratory rate	2
	Persistent breathing distress, wheezing	3
Coat / Skin	Smooth, flat coat	0
	Slightly roughened coat, lack of grooming, slight reddening, or edema at vaccination site	1
	Rough, dull looking coat, moderate reddening, or edema at vaccination site	2
Eyes	Eyes bright and clear	0
	Clear discharge from eyes / nose	1
	Conjunctivitis, yellow discharge from eyes and nose	2
Appetite/drinking	Normal, eats most of food offered	0
	Little food eaten, slight interest in treats	1
	Shows no interest in food or treats, nothing eaten or drunk	2
Temperature	Less than 1°C (+/−) change from baseline	0
	1.0°C to 1.5°C increase from baseline	1
	More than 1.6°C to 2°C increase from baseline	2
	More than 2°C increase from baseline, more than 42°C or more than 2°C decrease within 24h	3
Total clinical score (out of a theoretical score of 21)		

*The individual scores and total score for all 8 parameters are recorded daily per animal. Humane Endpoints are based on these scores.

Appendix Table 2. Clinical evaluation of individual or combined scores for ferrets*

Clinical Assessment	Mild	Moderate	Moderate†	Severe††
Total Clinical Score*	1–4	5–10	11–15	16 or more
Maximum for any one parameter*	1–2	1–2	3	3

*Humane Endpoints are based on these scores.
†Moderate severity is recorded if intervention results in improvement and reduction in clinical score after 4h. The humane endpoint is reached, requiring euthanasia, if no improvement occurs after 4h.
††Severe humane endpoint requires euthanasia.

Appendix Table 3. Clinical scoring scheme for pigs infected with influenza A viruses

Parameter	Criteria	Score*
Alertness	Attentive (curious, alert)	0
	Slightly reduced – hesitant, disinterested	1
	Inactive, gets up only when stimulated, lies down again	2
	Recumbent, does not get up when stimulated	3
Body shape/posture	Relaxed, full stomach, ‘round’ body	0
	Hunched back, empty stomach, thinned body muscles	2
	Flaccid or hunched and rigid body, emaciated, ribs and backbone showing	3
Breathing	Normal	0
	Sneezing / coughing	1
	Intermittent breathing difficulties, or increase/decrease respiratory rate	2
	Persistent breathing distress, wheezing	3
Coat / Skin	Evenly light pink skin, hair coat flat	0
	Reddened or pale skin, local swelling at injection site	1
Eyes / nose	Eyes bright and clear, light pink conjunctiva	0
	Clear discharge from eyes / nose, reddened eyes	1
	Yellow / green discharge from eyes / nose	2
Appetite	Greedy, hungry, all food finished	0
	Eats slowly when fed, some food remaining	1

Parameter	Criteria	Score*
	Does not eat when fed but tastes food. Food only partially eaten.	2
	Shows no interest in food, nothing eaten/drunk.	3
Defecation	Soft feces, normal amount	0
	Loose feces	1
	Diarrhea	2
Temperature	37.0°C – 39.4°C (normal range 38.7–39.8)	0
	39.5°C – 39.9°C	1
	40.0°C – 40.9°C or less than 37°C	2
	41.0°C or above	3
Total clinical score (out of a theoretical score of 20)		

*The individual scores and total score for all 8 parameters are recorded daily per animal. Humane Endpoints are based on these scores.

Appendix Table 4. Clinical evaluation of individual or combined scores for pigs*

Clinical Assessment	Mild	Moderate	Moderate†	Severe††
Total Clinical Score*	1–4	5–7	8	9 or more
Maximum for any one parameter*	1	1–2	3	3

*Humane Endpoints are based on these scores.
†Moderate severity is recorded if intervention results in improvement and reduction in clinical score after 4h. The humane endpoint is reached, requiring euthanasia, if no improvement occurs after 4h.
††Severe humane endpoint requires euthanasia.

References

1. Karakus U, Crameri M, Lanz C, Yángüez E. Propagation and titration of influenza viruses. In: Yamauchi Y, editor. Influenza virus: methods and protocols. New York, NY: Springer New York; 2018. p. 59–88.

2. Rovida F, Piralla A, Marzani FC, Moreno A, Campanini G, Mojoli F, et al. Swine influenza A (H1N1) virus (SIV) infection requiring extracorporeal life support in an immunocompetent adult patient with indirect exposure to pigs, Italy, October 2016. Euro Surveill. 2017;22:30456. **PMID 28183395**

3. Brookes SM, Núñez A, Choudhury B, Matrosovich M, Essen SC, Clifford D, et al. Replication, pathogenesis and transmission of pandemic (H1N1) 2009 virus in non-immune pigs. PLoS One. 2010;5:e9068. PubMed https://doi.org/10.1371/journal.pone.0009068

4. Everett HE, Nash B, Londt BZ, Kelly MD, Coward V, Nunez A, et al. Interspecies transmission of reassortant swine influenza A virus containing genes from swine influenza A(H1N1)pdm09 and A(H1N2) viruses. Emerg Infect Dis. 2020;26:273–81. PubMed https://doi.org/10.3201/eid2602.190486

5. Gauger PC, Vincent AL. Serum virus neutralization assay for detection and quantitation of serum neutralizing antibodies to influenza A virus in swine. Methods Mol Biol. 2020;2123:321–33. PubMed https://doi.org/10.1007/978-1-0716-0346-8_23

6. Spencer AJ, Hill F, Honeycutt JD, Cottingham MG, Bregu M, Rollier CS, et al. Fusion of the Mycobacterium tuberculosis antigen 85A to an oligomerization domain enhances its immunogenicity in both mice and non-human primates. PLoS One. 2012;7:e33555. PubMed https://doi.org/10.1371/journal.pone.0033555

7. Löndt BZ, Brookes SM, Nash BJ, Núñez A, Stagg DA, Brown IH. The infectivity of pandemic 2009 H1N1 and avian influenza viruses for pigs: an assessment by ex vivo respiratory tract organ culture. Influenza Other Respir Viruses. 2013;7:393–402. PubMed https://doi.org/10.1111/j.1750-2659.2012.00397.x
